# RESTORATION OF HYPOXIA SIGNALING IMPROVES AGING-ASSOCIATED LOSS OF SKELETAL MUSCLE REGENERATIVE POTENTIAL

**DOI:** 10.1093/geroni/igz038.2679

**Published:** 2019-11-08

**Authors:** Kodi Udeh, Bin Li, Yori Endo, Adriana Panayi, Dharaniya Sakthivel, Ron Neppl, Amy Wagers, Idranil Sinha

**Affiliations:** 1 Brigham and Women’s Hospital, Harvard Medical School, Boston, Massachusetts, United States; 2 Brigham and Women's Hospital, Division of Plastic Surgery, Boston, Massachusetts, United States; 3 Harvard Stem Cell Institute, Cambridge, Massachusetts, United States

## Abstract

Skeletal muscle retains the ability to regenerate throughout life, but this decreases significantly with aging. The present study investigates whether aging-associated loss of muscle hypoxia signaling limits regenerative potential. Utilizing young (3 months) and old (22-24 months) mice, skeletal muscle from old mice exhibited a 40% decline in the cross-sectional area (CSA) of newly regenerating fibers following cryoinjury at day 10 (p < 0.01) post-injury as compared to young. Focused PCR array demonstrated a greater than 3-fold decline in expression of the majority of hypoxia signaling genes. In particular, aryl hydrocarbon receptor nuclear translocator (ARNT), which is required for downstream hypoxia signaling and the transcription of hypoxia response genes, is 5-fold lower for both gene expression (p < 0.01) and protein levels (p < 0.01) in old versus young mice. To determine the effects of ARNT on muscle regeneration, we utilized a genetically modified mouse which results in an 80% decrease in ARNT gene expression following activation, specifically in skeletal muscle. Compared to littermate controls, mice with a muscle specific knockdown of ARNT (mKO ARNT) exhibit a 30% decline in regenerating fiber sizes at day 10 (p < 0.01) following cryoinjury, without any loss of regenerative potential in FACS isolated satellite cells ex vivo. Administration of a pharmacologic hypoxia activator, ML228, induced a 30% increase in regenerating fiber CSA in both old mice and mKO ARNT mice (p < 0.01) as compared to treatment with vehicle control. These data suggest hypoxia signaling declines with aging in skeletal muscle and activation of hypoxia signaling may promote regeneration.

